# Brainstem NTCP and Dose Constraints for Carbon Ion RT—Application and Translation From Japanese to European RBE-Weighted Dose

**DOI:** 10.3389/fonc.2020.531344

**Published:** 2020-11-24

**Authors:** Jon Espen Dale, Silvia Molinelli, Barbara Vischioni, Viviana Vitolo, Maria Bonora, Giuseppe Magro, Andrea Mairani, Azusa Hasegawa, Tatsuya Ohno, Olav Dahl, Francesca Valvo, Piero Fossati

**Affiliations:** ^1^ Department of Clinical Science, Faculty of Medicine, University of Bergen, Bergen, Norway; ^2^ Department of Oncology and Medical Physics, Haukeland University Hospital, Bergen, Norway; ^3^ National Center of Oncological Hadrontherapy, Pavia, Italy; ^4^ Heidelberg Ion-Beam Therapy Center, Heidelberg, Germany; ^5^ Osaka Heavy Ion Therapy Center, Osaka, Japan; ^6^ Department of Radiation Oncology, Gunma University Graduate School of Medicine, Gunma, Japan; ^7^ MedAustron Ion Therapy Center, Wiener Neustadt, Austria

**Keywords:** carbon ion radiotherapy, normal tissue complication probability, dose constraints, local effect model, microdosimetric kinetic model, relative biological effectiveness (RBE), brainstem tolerance

## Abstract

**Background and Purpose:**

The Italian *National Center of Oncological Hadrontherapy* (CNAO) has applied dose constraints for carbon ion RT (CIRT) as defined by Japan’s *National Institute of Radiological Sciences* (NIRS). However, these institutions use different models to predict the *relative biological effectiveness* (RBE). CNAO applies the *Local Effect Model I* (LEM I), which in most clinical situations predicts higher RBE than NIRS’s *Microdosimetric Kinetic Model* (MKM). Equal constraints therefore become more restrictive at CNAO. Tolerance doses for the brainstem have not been validated for LEM I-weighted dose (*D*_LEM I_). However, brainstem constraints and a *Normal Tissue Complication Probability* (NTCP) model were recently reported for MKM-weighted dose (*D*_MKM_), showing that a constraint relaxation to *D*_MKM|0.7 cm^3^_ <30 Gy (RBE) and *D*_MKM|0.1 cm^3^_ <40 Gy (RBE) was feasible. The aim of this work was to evaluate the brainstem NTCP associated with CNAO’s current clinical practice and to propose new brainstem constraints for LEM I-optimized CIRT at CNAO.

**Material and Methods:**

We reproduced the absorbed dose of 30 representative patient treatment plans from CNAO. Subsequently, we calculated both *D*_LEM I_ and *D*_MKM_, and the relationship between *D*_MKM_ and *D*_LEM I_ for various brainstem dose metrics was analyzed. Furthermore, the NTCP model developed for *D*_MKM_ was applied to estimate the NTCPs of the delivered plans.

**Results:**

The translation of CNAO treatment plans to *D*_MKM_ confirmed that the former CNAO constraints were conservative compared with *D*_MKM_ constraints. Estimated NTCPs were 0% for all but one case, in which the NTCP was 2%. The relationship *D*_MKM_/*D*_LEM I_ could be described by a quadratic regression model which revealed that the validated *D*_MKM_ constraints corresponded to *D*_LEM I|0.7 cm^3^_ <41 Gy (RBE) (95% CI, 38–44 Gy (RBE)) and *D*_LEM I|0.1 cm^3^_ <49 Gy (RBE) (95% CI, 46–52 Gy (RBE)).

**Conclusion:**

Our study demonstrates that RBE-weighted dose translation is of crucial importance in order to exchange experience and thus harmonize CIRT treatments globally. To mitigate uncertainties involved, we propose to use the lower bound of the 95% CI of the translation estimates, *i.e.*, *D*_LEM I|0.7 cm^3^_ <38 Gy (RBE) and *D*_LEM I|0.1 cm^3^_ <46 Gy (RBE) as brainstem dose constraints for 16 fraction CIRT treatments optimized with LEM I.

## Introduction

There is an increasing interest in using carbon ion radiotherapy (CIRT) for the treatment of advanced, radioresistant tumors. The physical properties of CIRT allow for delivering a high dose to the tumor, while the finite distal depth dose and sharp lateral penumbra can be utilized to spare nearby organs at risk (OARs) from excessive dose. Furthermore, carbon ions exhibit high linear energy transfer (LET) properties, which lead to more efficient cell killing (higher relative biological effectiveness (RBE)), compared with photon and proton RT. However, there are substantial uncertainties regarding the clinical RBE of carbon ions. Therefore, prescription doses, tolerance doses to OARs, and normal tissue complication probability (NTCP) models based on experience with photon or proton RT may not be applicable to CIRT and should preferably be derived from CIRT data.

Two major approaches have been used for the clinical implementation of CIRT. Spearheaded by the National Institute of Radiological Sciences (NIRS), Chiba, Japan, the Japanese centers are using hypofractionated treatment schedules (16 fractions of 3.6–4.6 Gy (RBE)) in which prescription doses and OAR tolerance doses initially were defined through carefully conducted dose-escalation trials. Originally, the *mixed beam model* (1) was developed to predict the RBE of the passively scattered carbon ion beams with *tumor response* as the relevant endpoint. Later, with the implementation of scanned beam delivery, the *modified microdosimetric kinetic model* (MKM) (2–5) was introduced. Since these two models have been validated for consistency, they are hereby collectively abbreviated as MKM.

In contrast, CIRT at the Gesellschaft für Schwerionenforschung (GSI), Darmstadt, Germany, was initiated with moderately hypofractionated schedules (20–22 fractions of 3.0–3.5 Gy (RBE)) in which the *Local Effect Model Version I* (LEM I) (6, 7) was used to predict the RBE of CIRT for *late responding normal tissues* (*i.e.*, central nervous system tissue). Trusting the LEM I to be sufficiently accurate, dose constraints derived from photon RT could be applied for CIRT treatments. An additional assumption for this approach was that the linear quadratic (LQ) formalism was applicable also for CIRT.

When the *National Center of Oncological Hadrontherapy* (CNAO, Italy) (8) started treating patients with LEM I-optimized CIRT in 2012, the successful treatment approach developed at NIRS was adopted. However, comparative studies show that the LEM I predicts a 5–15% higher RBE in the spread out Bragg peak (SOBP) of a carbon ion beam, relative to the MKM (9, 10). In the entrance region, the RBE predicted by LEM I can be 60% higher (11). Consequently, dependent on the clinical indication, prescription doses at CNAO (reported in LEM I-weighted dose, *D*_LEM I_) were increased 5–15% relative to the prescription doses at NIRS (reported in MKM-weighted dose (*D*_MKM_)) (9, 10). In contrast, dose constraints to OARs were not adjusted. This was a cautious approach mitigating various uncertainties related to the adaptation of NIRS prescription doses (*i.e.*, differences in RBE model, beam delivery method, dose optimization process, *etc.*).

For the brainstem, the dose constraint at CNAO was therefore set to be <30 Gy (RBE) to no more than 1% of the organ’s volume (*D*_LEM I│1%_), following the tradition of NIRS (12). Since this constraint becomes more restrictive in LEM I-optimized CIRT, CNAO has so far treated more than 1,000 patients with advanced tumors in the head and neck region (for example, skull base, nasopharynx, and sinonasal sites) without experiencing any grade of radiation-induced brainstem injury. Thus, the constraint needs to be updated to provide optimal treatments in cases where the target volume is located close to the brainstem. However, it is challenging to propose new and reasonable constraints since no toxic events have been reported from any institution applying LEM I-weighted doses for CIRT.

Recently, a dose-response analysis of brainstem toxicity following *D*_MKM_-optimized CIRT at *Gunma University Heavy Ion Medical Center* (GHMC) (13) was published by Shirai et al. (14). None of the 85 patients included in this analysis experienced symptomatic brainstem toxicity. However, four cases of focal brainstem contrast enhancement were detected on routine magnetic resonance imaging (MRI) during follow-up. This was defined as central nervous system (CNS) necrosis grade 1 events according to the *Common Terminology Criteria for Adverse Events version 4.0* (CTCAE). Even these asymptomatic events did not occur before the maximum dose (*D*_MKM|max_) exceeded 48 Gy (RBE), showing that current constraint may be conservative even when applied for *D*_MKM_. The brainstem volume receiving more than 30 Gy (RBE) (*V*_30 Gy (RBE)_) and 40 Gy (RBE) (*V*_40 Gy (RBE)_) were independent risk factors for this endpoint. Brainstem toxicity of any grade did not occur before *V*_30 Gy (RBE)_ exceeded 0.7 cm^3^ and *V*_40 Gy (RBE)_ exceeded 0.1 cm^3^. Since these values relate to radiologically detectable, but asymptomatic alterations in the brainstem, they may serve as constraints to avoid symptomatic injury. Shirai et al. also fitted their data to the Lyman-Kutcher-Burman (LKB) NTCP model (15–17), resulting in the following model parameters: volume-effect parameter (*n*) = 0.08, biodiversity parameter (*m*) = 0.08, and the *equivalent uniform dose* (EUD) corresponding to 50% probability of toxicity (TD50) = 32.4 Gy (RBE).

The goal of this work is therefore to:

1. evaluate the brainstem NTCP associated with CNAOs current clinical practice by applying the NTCP model published by Shirai et al.
2. convert the *D*_MKM_ validated constraints into *D*_LEM I_, providing guidance for the proposal of new dose constraints to be used at CNAO and other centers applying LEM I.

## Material and Methods

### Treatment Plan Selection and CIRT at CNAO

The dose distributions of 30 CIRT treatments with target volumes close to the brainstem were included in this study. Details on disease site, histology, and prescription dose are presented in **Table 1**. The treatments were given at CNAO in the period 2013–2014 as part of prospective protocols (CNAO S9/2012/C, CNAO S12/2012/C, and CNAO S15/2012/C) approved by the Regional Ethics Committee. Signed consent was required for participation. The plans were optimized for a prescribed *D*_LEM I_ of 68.8–76.8 Gy (RBE) in 16 fractions (4 fractions/week) using the *syngo^®^* RT Planning (Siemens Healthcare, Erlangen, Germany) treatment planning system (TPS). Dose constraint for the brainstem was *D*_LEM I│1%_ ≤30 Gy (RBE). Additionally, a constraint of *D*_LEM I│1%_ ≤35 Gy (RBE) was applied to a 3-mm planning OAR volume (PRV) for plan optimization purposes.

**Table 1:** Disease and treatment characteristics.

Case nr.	Histology	Site	Total *D*_LEM I_ (Gy (RBE))	Fraction *D*_LEM I_ (Gy (RBE))
1	Chordoma	Skull base	70.4	4.4
2	Mesenchymal tumor	Frontal sinus	76.8	4.8
3	Chordoma	Skull base	70.4	4.4
4	Chordoma	Skull base	70.4	4.4
5	MPNST	Clivus	76.8	4.8
6	Chordoma	Skull base	70.4	4.4
7	ACC	Meckel’s cave	68.8	4.3
8	Chondrosarcoma	Nasal cavity	70.4	4.4
9	Chordoma	Clivus	70.4	4.4
10	Chordoma	Clivus	70.4	4.4
11	Chordoma	Clivus	70.4	4.4
12	ACC	Maxillary sinus	68.8	4.3
13	Chordoma	Clivus	70.4	4.4
14	Chordoma	Clivus	70.4	4.4
15	Chondrosarcoma	Clivus	70.4	4.4
16	Chordoma	Skull base	70.4	4.4
17	ACC	Maxillary sinus	68.8	4.3
18	ACC	Nasopharynx	68,8	4,3
19	Chordoma	Clivus	70.4	4.4
20	Chondrosarcoma	Skull base	70.4	4.4
21	Cordoma	Clivus	70.4	4.4
22	ACC	Maxillary sinus	68.8	4.3
23	ACC	Skull base	68.8	4.3
24	Chordoma	Clivus	70.4	4.4
25	Pleomorphic sarcoma	Clivus	76.8	4.8
26	ACC	Paranasal sinuses	68.8	4.3
27	Chordoma	Clivus	70.4	4.4
28	Acinar cell carcinoma	Ethmoid/nasal cavity	68.8	4.3
29	ACC	Maxillary sinus	68.8	4.3
30	Chordoma	Clivus	70.4	4.4

MPNST, Malignant peripheral nerve sheath tumor; ACC, Adenoid cystic carcinoma.

In general, the strategy to obtain a robust treatment plan is similar at CNAO and GHMC: Multiple beam angles (3 to 4), dominantly originating from the horizontally fixed beam line, are achieved by couch rotation and/or by multiple immobilization positions where the patient’s head is positioned either straight or rotated. Due to particle range uncertainty, beam angles are chosen so that most of the dose to the brainstem originates from the beam’s sharp lateral penumbra, rather than the distal dose fall-off. Beams traversing through the brainstem are never used.

### Recalculation of RBE-Weighted Dose Distributions

The patients’ computed tomography (CT) image files, structure set files, dose files, and plan files were exported from the *syngo^®^* TPS and imported to the *matRad* open source multimodality radiation TPS (https://e0404.github.io/matRad/) (18) in which the absorbed dose (*D*_Abs_) and *D*_LEM I_ were reproduced. The input parameters used clinically for LEM I were applied, *i.e.*, *α_γ_* = 0.1 Gy^−1^, *β_γ_* = 0.05 Gy^−2^, *D_t_* = 30 Gy, *s*_max_ = 3.1 Gy^−1^, *R_n_* = 5 μm (7). The DVHs of targets and OARs were compared with the corresponding DVHs of the dose distribution from the *syngo^®^* TPS to ensure correct reproduction of both *D*_Abs_ and *D*_LEM I_ (results not reported). Secondly, MKM was implemented in the matRad TPS code using the input parameters used clinically (*R_d_* = 0.32 µm, *R_n_* = 3.9 µm, *α*_0_ = 0.172 Gy^−1^, *β* = 0.0615 Gy^−2^, *α_r_* = 0.764 Gy^−1^, *F*_Clin_ = 2.39) (2, 11) and *D*_MKM_ was derived from the exact same absorbed dose and LET spectra. This enabled a direct comparison of each patient’s *D*_LEM I_ and *D*_MKM_ based exclusively on the differences in the RBE modeling.

### Estimation of Brainstem NTCP

Using the *D*_MKM_ distributions, the brainstem NTCP for each treatment plan was calculated by the LKB method, using the model parameters suggested by Shirai et al. (14): *n* = 0.08, *m* = 0.08, and TD50 = 32.4 Gy (RBE).

### RBE-Weighted Dose Translation

For each brainstem, the *D*_MKM|0.7 cm^3^_ and *D*_MKM|0.1 cm^3^_ were plotted as a function of *D*_LEM I|0.7 cm^3^_ and D_LEM I|0.1 cm^3^_, respectively. A curve fitting procedure was performed with the software IBM SPSS Statistics for Windows, version 24.0 (IBM Corp., Armonk, NY, U.S.A.) in order to produce a dose translation model.

### Verification of Dose Translation Model

As a last step, we wanted to verify that the dose translation model correctly predicted the *D*_LEM I_/*D*_MKM_ relationship also for higher brainstem doses than our original data. Therefore, five treatment plans, in which the original *D*_LEM I_ constraint caused suboptimal dose coverage to the clinical target volume (CTV *D*_95%_ <95% of prescription dose), were reoptimized applying a new set of *D*_LEM I_ constraints as proposed by this work (see “**RESULTS**”). Subsequently, these new plans were recalculated to *D*_MKM_. These procedures, which were conducted exclusively to confirm the relationship of the RBE models, were performed with the RayStation^®^ 6.99 TPS (RaySearch Laboratories AB, Stockholm, Sweden), where both the LEM I and MKM were implemented with the respective model input parameters as mentioned earlier.

## Results

**Figure 1:**
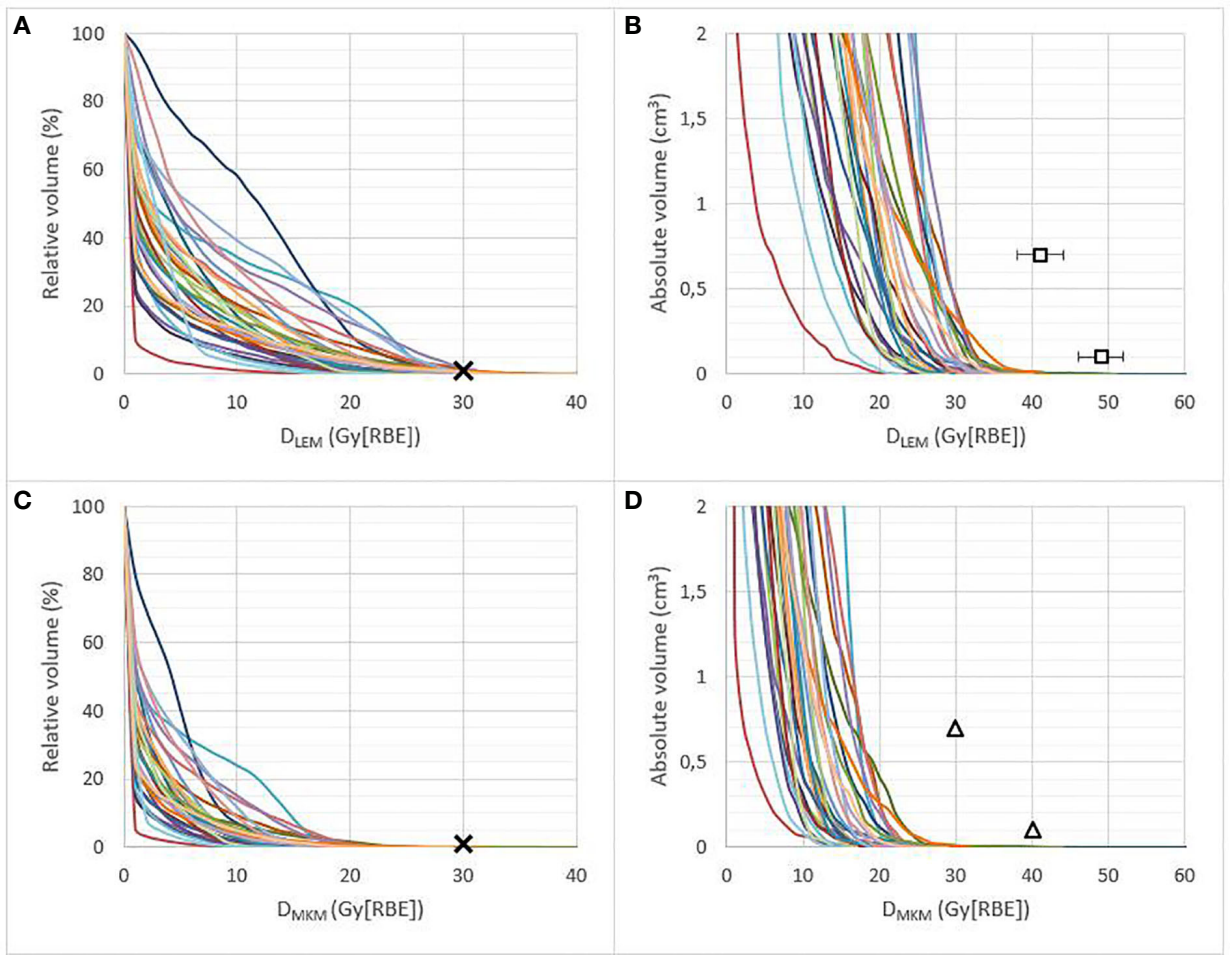
Brainstem DVHs in relative **(A, C)** and absolute volume (≤2 cm^3^) **(B, D)** of 30 patients treated at CNAO, presented in *D*_LEM I_ **(A, B)** and *D*_MKM_ **(C**, **D)**. Crosses represent the former CNAO and NIRS dose constraint of *D*_1%_ ≤30 Gy (RBE). Triangles represent the new *D*_MKM_ constraints *V*_40 Gy (RBE)_ <0.1 cm^3^ and *V*_30 Gy (RBE)_ <0.7 cm^3^ as defined by Shirai et al. (14). Squares in **(B)** represent the possible new *D*_LEM I_ constraints (error bars, 95% CI) resulting from the dose translation model presented in this work, see **Figure 3**.

Brainstem DVHs in relative and absolute volumes are presented in both D_LEM I_ and *D*_MKM_ in **Figure 1**, showing the substantial decrease in RBE-weighted doses when the MKM is applied as RBE model.

The median brainstem *D*_LEM I|1%_ was 23.7 Gy (range, 11.2–31.3 (RBE)), which corresponded to only 12.4 Gy (range, 5.5–21.8 (RBE)) in *D*_MKM_, highlighting the restraining effect of the original CNAO constraint in achieving optimal CIRT treatments.

**Figure 2:**
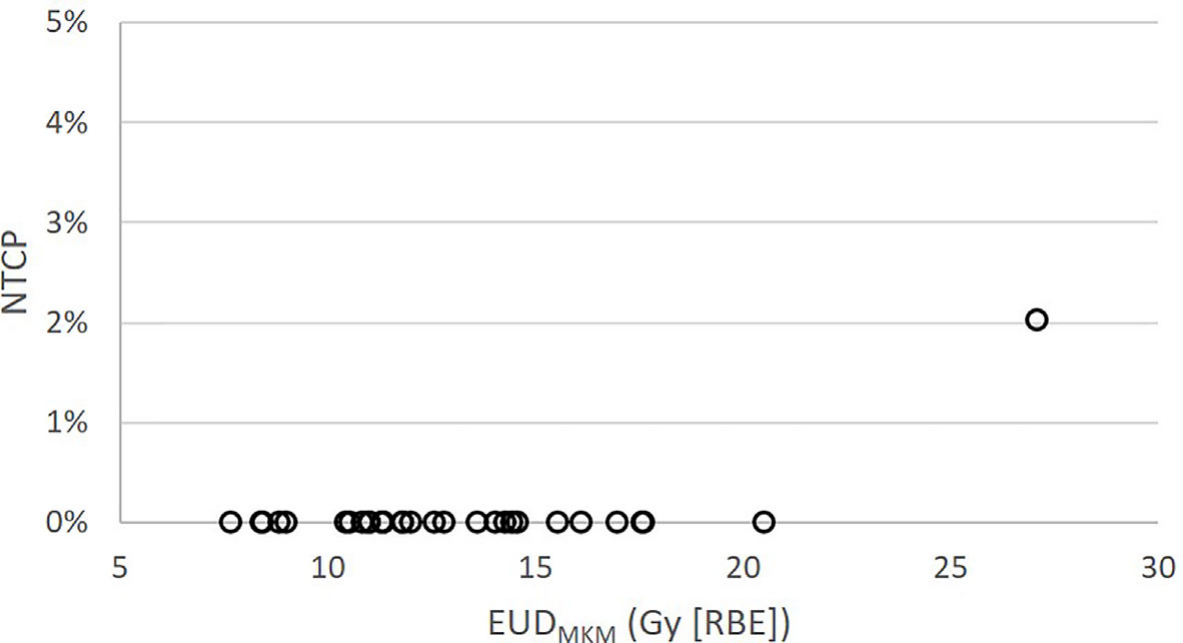
Brainstem NTCP for the 30 patients treated at CNAO as function of EUD_MKM_ according to the NTCP model published by Shirai et al. (14).

Only four of the brainstems received *D*_MKM_ *>*30 Gy (RBE), each of them to a volume smaller than 0.05 cm^3^. As seen in **Figures 1B, D**, the highest *D*_LEM I_ to the brainstem volumes 0.7 and 0.1 cm^3^ were 29 Gy (RBE) and 35 Gy (RBE), respectively, corresponding to 17 Gy (RBE) and 25 Gy (RBE) in *D*_MKM_. These modest doses resulted in a very low probability of asymptomatic (grade 1) brainstem injury according to the NTCP model published by Shirai et al. (14): One patient had an NTCP of 2%, while the NTCPs of the remaining 29 patients were close to 0%, see **Figure 2**.

**Figure 3:**
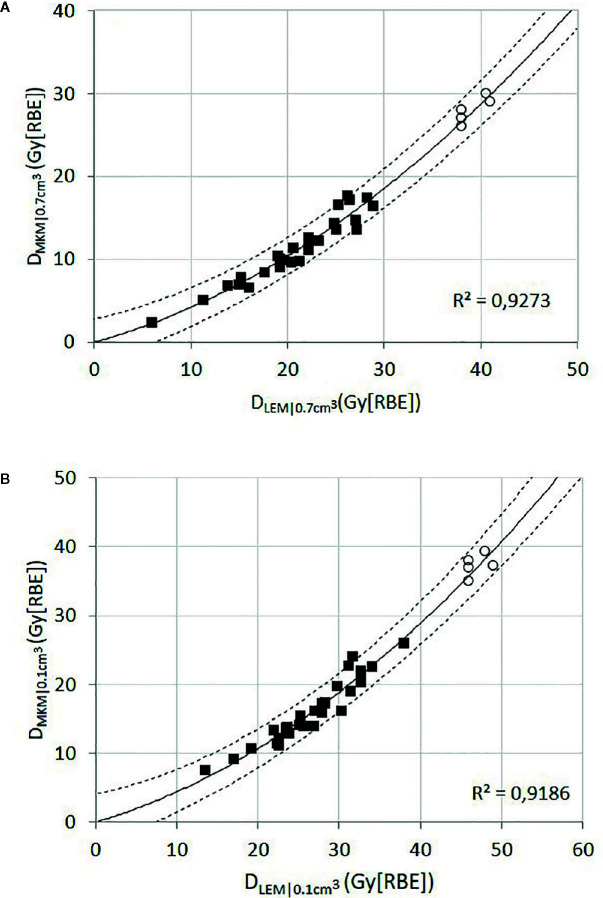
Black squares represent the relationship of *D*_LEM I_ to *D*_MKM_ for the dose metrics D_0.7 cm^3^_ **(A)** and *D*_0.1 cm^3^_ **(B)** for each individual brainstem. The solid line represents the quadratic function providing the best fit to the data points (black squares), assuming that the intercept should be in the origin. The dashed lines represent the 95% CI. The open circles represent the data collected from the reoptimized plans; these data points were not used for the curve fitting procedure.

For each patient, the brainstem dose metrics *D*_LEM I|0.7 cm^3^_ and *D*_LEM I|0.1 cm^3^_ were plotted against the corresponding dose metric in *D*_MKM_ (**Figure 3**). With the assumption that the intercept should be at origin (*D*_LEM_ = 0 Gy (RBE) when *D*_MKM_ = 0 Gy (RBE)), we found that the quadratic regression model

$$D_{MKM}=(b1\times D_{LEM\;I})+(b2\times {\left[D_{LEM\;I}\right]}^{2})$$

adequately fit both sets of data (coefficients of determination, *R* ^2^ ≥ 0.918). Extrapolation of the models to the relevant dose levels revealed that a *D*_MKM|0.7 cm^3^_ of 30 Gy (RBE) and a *D*_MKM|0.1 cm^3^_ of 40 Gy (RBE) translates into a *D*_LEM I|0.7 cm^3^_ of 41 Gy (RBE) (95% CI, 38–44 Gy (RBE)) and a *D*_LEM I|0.1 cm^3^_ of 49 Gy (RBE) (95% CI, 46–52 Gy (RBE)), respectively.

**Figure 4:**
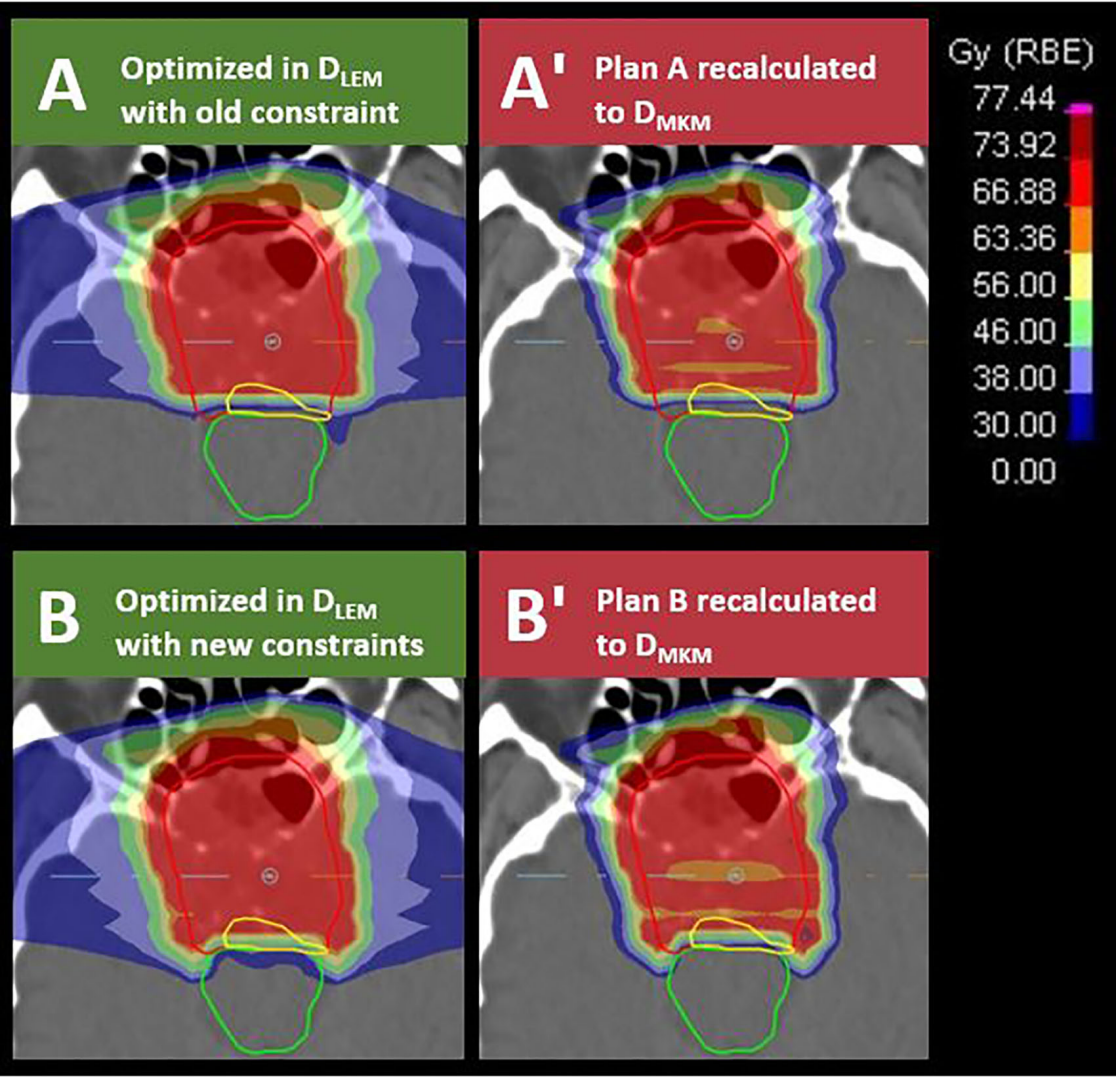
Transversal sections of *D*_LEM I_-optimized treatment plans applying brainstem (green contour) constraints of *D*_LEM I|1%_ <30 Gy (RBE) in plan **(A)** or *D*_LEM I|0.7 cm^3^_ <38 Gy (RBE) and *D*_LEM|0.1 cm^3^_ <46 Gy (RBE) in plan **B)**. The dose constraint levels are illustrated by dark blue, light blue, and light green isodose, respectively. Plans were subsequently recalculated to *D*_MKM_ **(A′, B′)**. Red isodose in plan **(A, B)** represents 95% of the target dose (70.4 Gy (RBE) in *D*_LEM I_). Note the improved dose coverage to the CTV (red contour) and to the part of the CTV in which the tumor recurred (yellow contour) in plan B compared with plan A. Dose to the brainstem remains compliant with the constraints defined by Shirai et al. when evaluated in *D*_MKM_ **(B′)**.

Subsequently, we reoptimized five of the treatment plans in which the old brainstem constraint (*D*_LEM I|1%_ <30 Gy (RBE)) caused suboptimal CTV dose coverage. For the reoptimization, new brainstem constraints within the lower half of the 95% CI of the dose translation estimates were applied, *i.e.*, *D*_LEM I|0.7 cm^3^_ <38–41 Gy (RBE) and *D*_LEM I| cm^3^_ <46–49 Gy (RBE). The relationship of *D*_LEM I_ to *D*_MKM_ for the dose metrics *D*_0.7 cm^3^_ and *D*_0.1 cm^3^_ from the reoptimized plans are plotted as open circles in the scatterplots of **Figure 3**. As can be seen, the values of these data pairs agree with the prediction of the dose translation model. To demonstrate the potential clinical impact of relaxing the constraints, a comparison of the original and reoptimized plans, displayed in both *D*_LEM I_ and *D*_MKM,_ is presented in **Figure 4**. For this patient, the proportion of the CTV receiving >95% of the prescription dose increased from 74 to 95%.

## Discussion

For the implementation of CIRT at CNAO, the goal has been to replicate the successful results achieved at Japanese CIRT centers, by translating NIRS prescription doses into equiefficient doses within the LEM I dose prescription system (9, 10). However, initially the OAR dose constraints were not adjusted correspondingly. This study clearly shows that the original brainstem dose constraint applied at CNAO is too conservative compared with the clinical practice in Japanese centers. In a recent publication on skull base chordomas treated at CNAO, Iannalfi et al. found that 92% of the local recurrences were attributable to suboptimal target dose in regions close to the brainstem or optic pathways (19). The estimated 5-year local control (LC) rate was 71%. This is inferior to the results reported by Japanese centers, where 5-year LC rates within the range 76–92% have been reported (20, 21).

Consequently, updated constraints for LEM I-optimized CIRT are urgently needed. In our opinion, due to the lack of publications addressing brainstem NTCP for LEM I-optimized CIRT, this aim was only achievable by making use of *D*_MKM_-validated dose constraints. Relating the CNAO DVHs to the new *D*_MKM_ constraints defined by Shirai et al. (**Figure 1D**) suggests that doses to the brainstem volumes 0.7 and 0.1 cm^3^ potentially could be increased by 13 Gy (RBE) and 15 Gy (RBE) in *D*_MKM_, respectively, compared with the former practice at CNAO. According to our dose constraint translation, the corresponding increase in *D*_LEM I_ would be approximately 12 Gy (RBE) (95% CI, 9–15 Gy (RBE)) and 14 Gy (RBE) (95% CI, 11–17 Gy (RBE)). This unveils an opportunity for improved target dose coverage, and thus improved treatment outcome, as demonstrated in **Figure 4**.

Recently, the European Particle Therapy Network (EPTN) released a consensus paper for dose constraints to various OARs (22), suggesting a general constraint of *D*_0.03 cm^3^_ ≤54 Gy (RBE) to the brainstem, with an option to allow for *D*_0.03 cm^3^_ ≤60 Gy (RBE) to the brainstem surface. Both constraints were expressed in *equivalent dose in 2 Gy fractions* (EQD2), with an assumed α/β ratio of 2 Gy. These guidelines are based on photon and proton RT toxicity data and are not necessarily applicable for CIRT due to the larger uncertainties involved in the prediction of the RBE. However, similar constraints are used for CIRT at the *Heidelberg Ion Beam Therapy Center* (HIT) in Germany (23), building on previous clinical experience of the GSI. Various publications from this institution explicitly report an absence of brainstem toxicity (24, 25). Consequently, these constraints are considered safe for CIRT under HIT’s current treatment paradigm, which consists of 20–22 fractions of 3.0–3.5 Gy (RBE) and 5–7 fractions per week. Although HIT also applies LEM I, these constraints may not be safely transferred to the 16 fraction/4 fractions per week treatment schedule of CNAO, as EQD2 conversion may not be sufficiently precise when fraction doses increase, due to uncertainties in the prediction of RBE.

**Figure 5:**
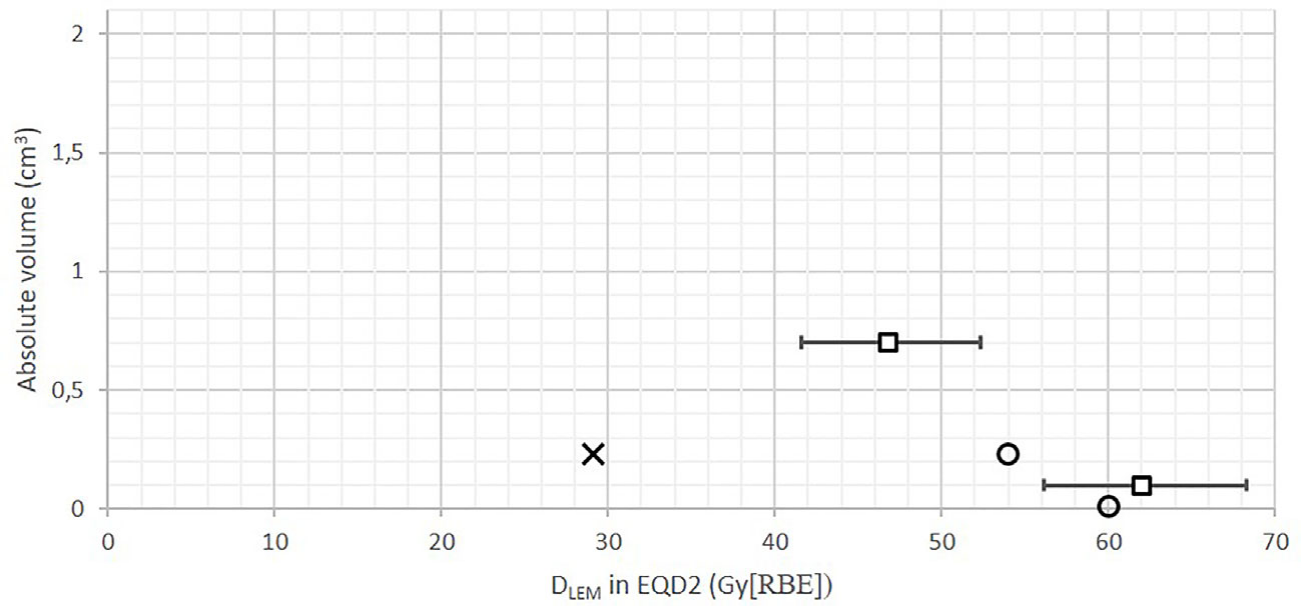
Absolute volume DVH showing old CNAO *D*_LEM I|1%_ <30 Gy (RBE) constraint (cross) and the translated Shirai constraints *D*_LEM I|0.7 cm^3^_ <41 Gy (RBE) and *D*_LEM I|0.1 cm^3^_ <49 Gy (RBE) (squares, error bars = 95% CI), converted into EQD2 (assuming α/β ratio = 2 Gy) in comparison with the EQD2 constraints applied at HIT as reported by Nikoghosyan et al. (21): *D*_LEM I|1%_ <54 Gy (RBE) and *D*_LEM I|max_ <60 Gy (RBE) (circles). As an approximation to the absolute volume relating to the *D*_1%_ constraints, the median brainstem volume in our data set (26 cm^3^) was used. The translated constraints are more closely related to the constraints used at HIT than the old CNAO constraint.

That being said, it is interesting to observe that our translated constraints, when converted into EQD2, relate closely to the EQD2 constraints used in clinical practice at HIT (23), see **Figure 5**.

**Figure 6:**
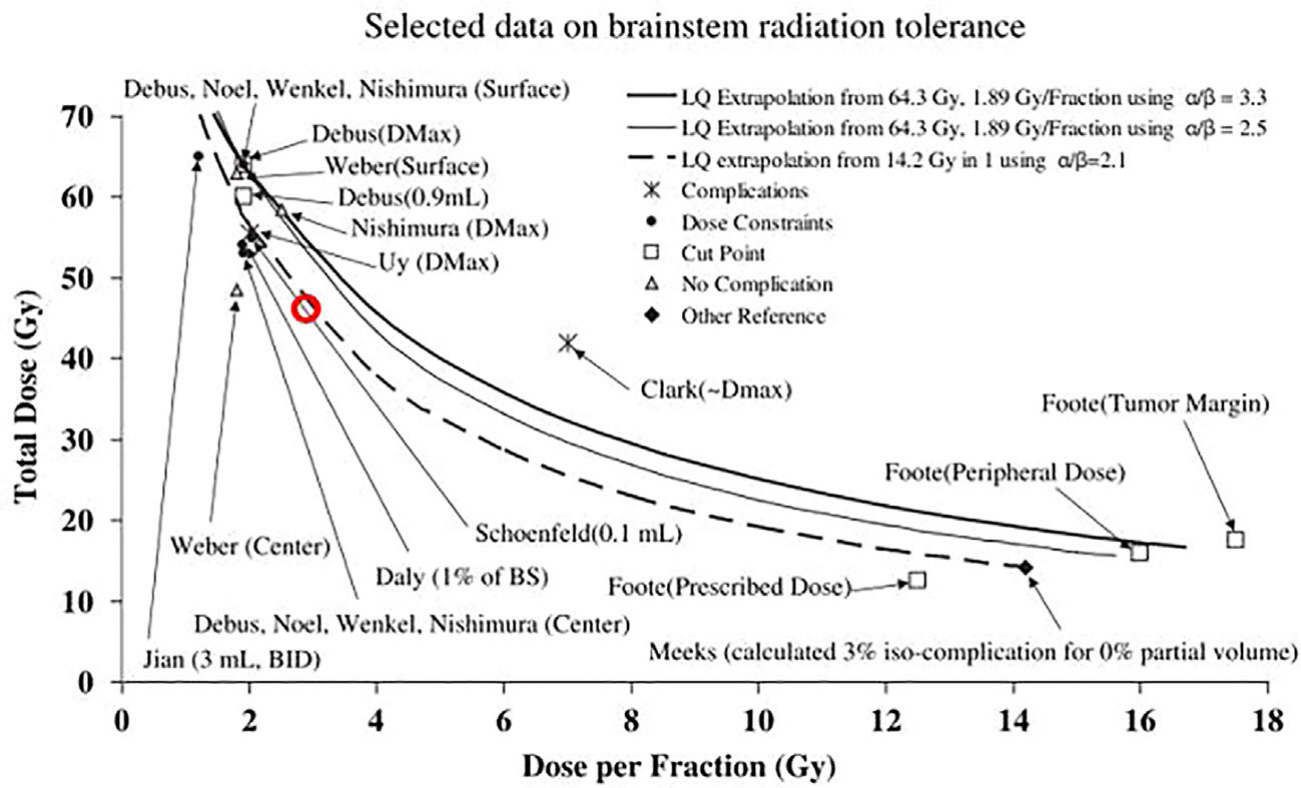
Figure 1 from Mayo et al. (23) reprinted with permission, comparing selected data on brainstem tolerance and dose constraints from stereotactic RT or normofractionated photon or proton RT, compared with the linear quadratic (LQ) model extrapolations. Data points are marked with the corresponding author and dose parameter considered in parenthesis. The *D*_LEM I|0.1 cm^3^_ <46 Gy (RBE) constraint for a 16-fraction LEM I-optimized CIRT, estimated by dose translation of the corresponding *D*_MKM_ constraint is superimposed as a red circle on the original figure.

In 2010, as part of the *Quantitative Analysis of Normal Tissue Effects in the Clinic* (QUANTEC) effort, brainstem constraints and tolerance doses following photon and proton RT were summarized in **Figure 1** in the organ-specific paper by Mayo et al. (26). Making use of the LQ model, tolerance doses from either normofractionated treatments or single fractionation stereotactic treatments were extrapolated to provide an approximation for the tolerance dose for hypofractionated treatments. The figure is reused in **Figure 6** of this paper, in which the *D*_LEM I|0.1 cm^3^_ constraint we derived from this work has been superimposed as a red circle. Clearly, our constraint complies with the projections of the LQ model, supporting the capacity of the LEM I to predict the RBE of CIRT for this endpoint with sufficient accuracy.

An advantage of our dose translation approach is that the fractionation regimen at GHMC is similar to that of CNAO, and therefore the uncertainty related to EQD2 conversion can be avoided. Furthermore, both GHMC and CNAO have adopted the traditions of NIRS, in regard to the choice of beam number, angles, and strategies to achieve a robust treatment plan. Lastly, both centers are restricted to the use of fixed beam lines, which inevitably restricts the freedom of beam angles and consequently favors harmonization of the treatments at the two centers additionally.

However, our method is affected by unavoidable uncertainties. Firstly, transferring dose constraints from a center with passive scattering beam delivery (PS) to a center with pencil beam scanning (PBS) may be controversial. The beam delivery techniques will inevitably cause differences in the radiation quality (mixture of primary and secondary particles and their corresponding LET values) of the beams, and the distribution and weighting of Bragg peaks may be very dissimilar. However, two studies have confirmed that the biological effect of the carbon ion beams of NIRS, HIT, and CNAO are identical (27, 28).

Secondly, the *D*_Abs_ underlying the RBE-weighted dose is calculated by different beam models at the two institutions. It has been shown that the *D*_Abs_ of a given RBE-weighted dose could on average vary about 2.5% in the *target region* of head and neck treatments, depending on the beam model (9). Differences related to beam modeling in the *out-of-target* areas have not been investigated, but one would expect to find more profound deviations in *D*_Abs_ especially within the lateral penumbra dose fall-off. This region is certainly sensitive to how the lateral spread of the beam is modeled. This is of importance, since the sharp lateral penumbra of the carbon ion beam typically is utilized to avoid high doses to the brainstem when it is located close to the tumor.

To conclude, these latter issues infer that the *D*_MKM_ that we reproduce in this work, based on the *D*_Abs_ of CNAO *D*_LEM I_-optimized treatment plans, are not an exact replica of GHMC treatment plans. Nevertheless, our dose translation approach definitely provides guidance as to how much the *D*_LEM I_ constraints at CNAO may be relaxed in order to match the Japanese constraints. As a measure of caution, we propose the lower bound of the 95% CI of the dose translation estimates, *i.e.*, *D*_LEM I|0.7 cm^3^_ <38 Gy (RBE) and *D*_LEM I|0.1 cm^3^_ <46 Gy (RBE), as possible brainstem constraints for LEM I-optimized CIRT in a 16-fraction schedule. These proposed constraint values imply *D*_LEM I_/*D*_MKM_ conversion factors of 1.27 and 1.15 for *D*_MKM_ fraction doses of 1.88 Gy (RBE) and 2.5 Gy (RBE) respectively, which is quite modest compared with the target dose conversion factors found by Steinsträter et al. (29), where conversion factors for the respective fraction doses were found to be >1.44 and >1.21.

Finally, as our conclusions rely on the results of Shirai et al., the limitations described in their study also apply to our work (small number of events, single institution study, *etc*.). Another essential assumption for the application of these constraints is that asymptomatic MRI contrast enhancement does not necessarily evolve into necrosis and therefore constraints that safeguard against this event most certainly will prevent the more meaningful clinical endpoint. Mere contrast enhancement is regarded as evidence of increased permeability of the blood-brain barrier (BBB), which results from radiation-induced alterations in endothelial and glial cell function (30). However, increased permeability does not necessarily lead to parenchymal damage as demonstrated for the spinal cord in a rat model (31). This phenomenon has also been documented for radiation-induced injury of the brain following CIRT, and it is hypothesized that since smaller volumes of CNS tissue is irradiated by particle therapy in comparison with photon RT, the probability of recovery will be higher (32). The observation that the lesions reported by Shirai et al. were reversible or stable in the absence of therapeutic intervention further supports the argument that no real necrosis had occurred.

In this setting, applying the CTCAE term *CNS necrosis grade 1* when only contrast enhancement is evident, as done by Shirai et al., may be confusing and potentially discourage physicians from referring patients to CIRT. However, the CTCAE lacks a proper predefined term to discriminate increased permeability in the BBB from a necrotic process. Moreover, neither the *SOMA-LENT scale* (subterm *MRI* in the *Analytic* scale) (33) nor the *RTOG/EORTC Late Morbidity Scoring Schema* (subterm *Brain*) (34) exhibit sufficient granularity to encompass this distinction. We therefore suggest to apply the CTCAE term *Nervous system disorders—Other*, and specifying it as *Brainstem reaction* as an analogy to the *Temporal lobe reaction* term coined by Gilman et al. (35), in which contrast enhancement would be a grade 1 “reaction,” thus avoiding the use of the misleading and more distressing term “necrosis.”

## Conclusions

Based on this work, these new constraints, *D*_LEM I|0.7 cm^3^_ <38 Gy (RBE) and *D*_LEM I|0.1 cm^3^_ <46 Gy (RBE), have been implemented in the prospective treatment protocols of CNAO since October 2018. They can serve as constraints also for other centers applying LEM I within CIRT schedules of 16 fractions. Indeed, these constraints have also been selected as the most optimal constraints available and have therefore recently been implemented in clinical practice at the MedAustron Ion Therapy Center (Wiener-Neustadt, Austria) for 16 fractions of CIRT treatment of skull base tumors optimized with LEM I.

This paper highlights a challenge that is unique for CIRT compared with other external beam RT modalities: the exchange of experience between Japanese and European CIRT facilities is severely hampered by the use of disparate RBE models. Fortunately, we anticipate that the recalculation of treatment plans to the alternative RBE model will become substantially less time consuming due to the introduction of such functionality in commercial TPSs. We therefore hope to see future CIRT publications reporting OAR toxicity, NTCP, and related dose metrics in both *D*_MKM_ and *D*_LEM I_, as our group recently has done for the optic nerve (36). This would accelerate the much needed validation of OAR constraints for both RBE models.

## Data Availability Statement

The datasets generated for this study are available on request to the corresponding author.

## Ethics Statement

The patients analyzed in the study cannot be identified as all the research has been conducted with anonymized data. All patients enrolled in the clinical trials at CNAO gave their free informed consent to the treatment and the use of their anonymized data for research purposes. The anonymized patient data used for this study originated from the clinical trials “CNAO S9/2012/C”, “CNAO S12/2012/C” and “CNAO S15/2012/C” approved by the CNAO Ethics Committee.

## Authors Contributions

The manuscript was mainly authored by JD with support from PF, SM, OD, TO, and BV. JD performed data analysis. Clinical treatment plans were optimized by medical physicists SM, GM, and AM with FV, MB, VV, BV, and PF as the responsible radiation oncologists. Clinical data were collected by VV, MB, BV, AH, and PF. JD, SM, and GM performed the recalculation of all treatment plans. FV, VV, and BV are responsible for the treatment protocols and analyzed patient follow-up together with MB and PF. JD, SM, OD, AM, TO, AH, and PF contributed to the development of the study methodology. All authors critically reviewed the manuscript. All authors contributed to the article and approved the submitted version.

## Funding

This work was supported by grants (grant no: BFS2015PAR02) from the Trond Mohn Foundation, Ytrebygdsvegen 215, Kokstad, Postboks 7150, 5020 BERGEN, Norway, tlf: +47 479 00 111, org.nr: 988 029 327.

## Conflict of Interest

The authors declare that the research was conducted in the absence of any commercial or financial relationships that could be construed as a potential conflict of interest.
